# Participant attention on the intervention target during repetitive passive movement improved spinal reciprocal inhibition enhancement and joint movement function

**DOI:** 10.1186/s40001-023-01418-7

**Published:** 2023-10-12

**Authors:** Ryo Hirabayashi, Mutsuaki Edama, Mai Takeda, Yuki Yamada, Hirotake Yokota, Chie Sekine, Hideaki Onishi

**Affiliations:** Institute for Human Movement and Medical Sciences, Niigata University of Health and Welfare, 1398 Shimami-cho, Kita-ku, , Niigata-shi, Niigata 950-3198 Japan

**Keywords:** H-reflex, Repetitive passive movement, Rehabilitation, Reciprocal inhibition, Electrical stimulation

## Abstract

This study aimed to evaluate the effects of the participant’s attention target during repetitive passive movement (RPM) intervention on reciprocal inhibition (RI) and joint movement function. Twenty healthy adults participated in two experiments involving four attention conditions [control (forward attention with no RPM), forward attention (during RPM), monitor attention (monitor counting task during RPM), ankle joint attention (ankle movement counting task during RPM)] during 10-min RPM interventions on the ankle joint. Counting tasks were included to ensure the participant’s attention remained on the target during the intervention. In Experiment 1, RI was measured before, immediately after, and 5, 10, 15, 20, and 30 min after the RPM intervention. In Experiment 2, we evaluated ankle joint movement function at the same time points before and after RPM intervention. The maximum ankle dorsiflexion movement (from 30° plantar flexion to 10° dorsiflexion) was measured, reflecting RI. In Experiment 1, the RI function reciprocal Ia inhibition was enhanced for 10 min after RPM under all attention conditions (excluding the control condition. D1 inhibition was enhanced for 20 min after RPM in the forward and monitor attention conditions and 30 min after RPM in the ankle joint attention condition. In Experiment 2, the joint movement function decreased under the forward and monitor attention conditions but improved under the ankle joint attention condition. This study is the first to demonstrate that the participant’s attention target affected the intervention effect of the RI enhancement method, which has implications for improving the intervention effect of rehabilitation.

## Background

Decreases in spinal reciprocal inhibition (RI) function are observed in patients with upper motor neuron disorders [1–3] and older adults [4–9]. Decreases in RI function cause excessive simultaneous activity between antagonistic muscles, impair smooth joint movement and walking function, and increase the risk of falls [10, 11].

In recent years, several studies have used techniques to enhance RI to inhibit excessive simultaneous muscle activity [12–23]. Approaches for enhancing RI include patterned electrical stimulation (PES) [15, 17, 21, 23, 24] and repetitive passive movement (RPM) [12, 13] peripheral stimulation, which show longer intervention after-effects than brain stimulation [14, 16, 18, 21]. In addition, in peripheral stimulation, compared to the after-effects at 10 min after a 20 min PES intervention, the effects of a 10 min RPM intervention persisted until 20 min after the intervention, indicating improved after-effects with a shorter intervention time [12, 13]. Furthermore, the combined use of brain stimulation [transcranial direct current stimulation (tDCS), intermittent theta burst transcranial magnetic stimulation (iTBS)] and peripheral stimulation synergistically improved the effects on RI after the intervention [21, 23]. RI was also enhanced by the combined use of motor imagery and peripheral electrical stimulation [25], indicating that primary motor cortex activity and increased excitability of the corticospinal tract enhanced the effects of peripheral stimulation.

Our research group focuses on simpler attention techniques than motor imagery training. The active attention process has been shown to play an important role in motor learning [26, 27] and motor performance [28, 29]. It has been reported that directing attention to behavior during a simple motor task activates the prefrontal area, cingulate cortex, supplementary motor area, premotor area, and cerebellum [30, 31] and contributes to the connection between the prefrontal and premotor areas [31]. It is recognized that motor-related areas are activated when attention is directed to the intervention target. [32] reported that attention to the index finger during movement attenuated short-interval intracortical inhibition (SICI) and increased corticospinal tract excitability.

Like brain stimulation and motor imagery, attention to the intervention target during RI enhancement may increase the intervention effect of peripheral stimuli. From previous studies [12, 13], we can expect to achieve enhanced intervention effects by directing attention to the intervention target during RPM, which as a method of RI augmentation can produce a large intervention effect in a short time. Examining the combined effects of RPM and attention to the intervention target will facilitate the development of more effective rehabilitation methods for clinical application. In addition, among the few reports on the post-intervention effect of the RPM RI enhancement method, only walking, balance function, and dynamic performance have been considered, and it is necessary to examine the intervention effect on a basic single joint movement. Because RI is the most important function during joint movement, we examined the intervention effect of the RI enhancement method on joint movement function.

Therefore, the purpose of this study was to examine the effect of RI enhancement by focusing attention on the target during the RPM intervention and evaluate the intervention effect via joint movement function. The hypothesis of this study is that attention to the intervention target during RPM intervention will inhibit SICI, increase corticospinal tract excitability, activate RI-inhibitory interneurons, and enhance RI potentiation. Moreover, we hypothesize that the joint movement function improvements will be modulated by RI enhancement and corticospinal tract excitability.

## Methods

We conducted two experiments to investigate how focusing attention on the target during RPM intervention affects RI and joint movement function. In Experiment 1, we set attention targets under four conditions during the RPM RI enhancement intervention and assessed RI before and after the intervention. In Experiment 2, the same four conditions were set, and the joint movement function was examined before and after the RPM intervention to examine the effect of the intervention.

### Study participants

Twenty healthy adults (age, 20.8 ± 0.9 years; height, 166.7 ± 7.8 cm; body weight, 57.5 ± 8.2 kg; 10 females) provided written informed consent to participate in this study. The study was approved by the Ethics Committee at the Niigata University of Health and Welfare (18,267–190,918). All experiments were conducted in accordance with the ethical standards of the Niigata University of Health and Welfare and the 1964 Helsinki Declaration and its later amendments.

### Experimental protocol overview

**Fig. 1:**
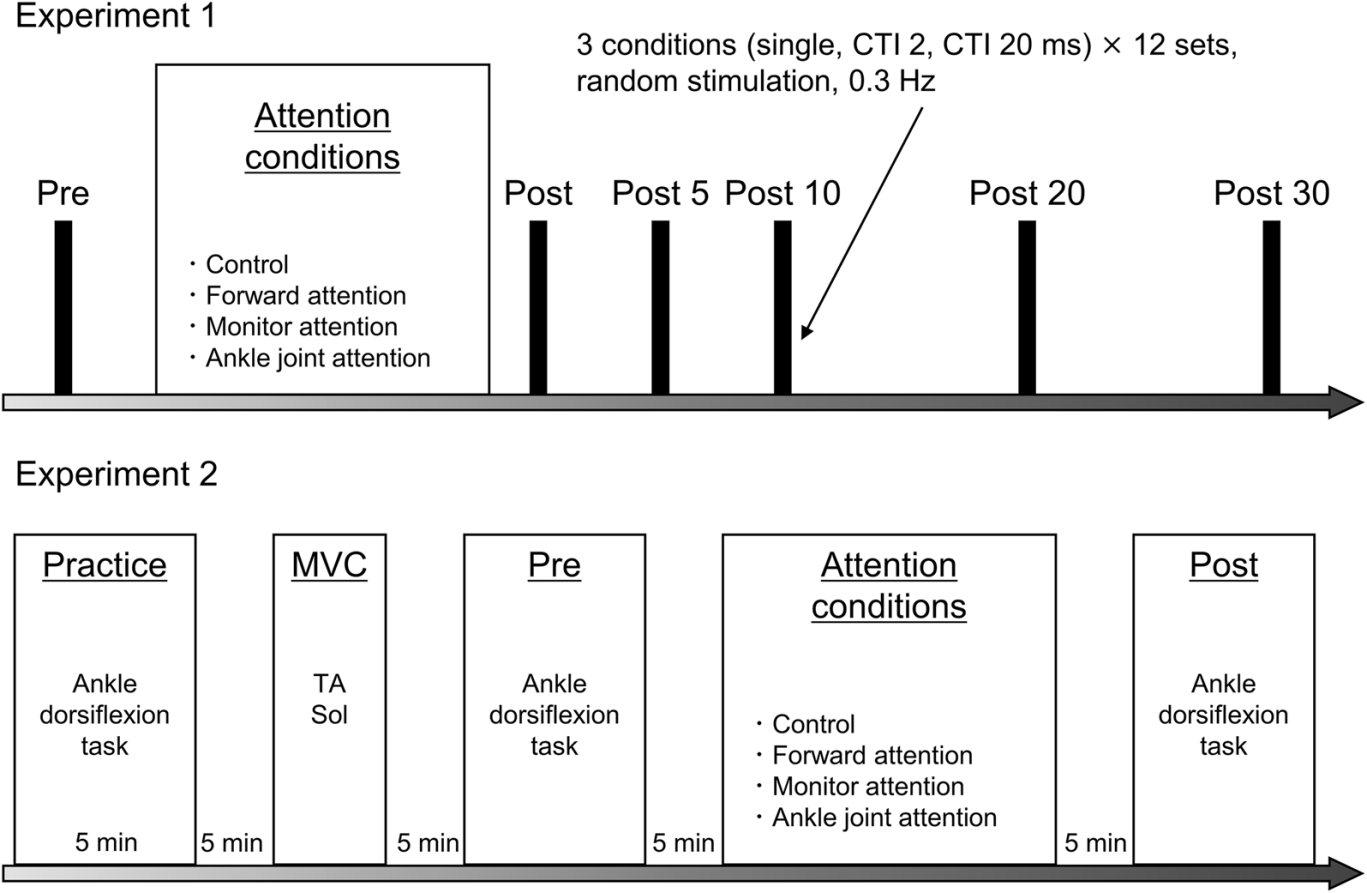
Experiment protocol. Four attention conditions, control, forward attention, monitor attention, and ankle joint attention, were randomly performed during the 10 min repetitive passive movement intervention. Experimental interventions were performed with an interval of at least 3 days between the conditions. In Experiment 1, the motor thresholds of the soleus muscle (Sol) maximum M wave amplitude and tibialis anterior muscle (TA) were measured before reciprocal inhibition (RI). RI was measured under three conditions [single, conditioning stimulation-test stimulation interval (CTI) 2 ms, CTI 20 ms]. The RI measurement was performed before the attention condition intervention (Pre), immediately after the intervention (Post), and 5 (Post 5), 10 (Post 10), 20 (Post 20), and 30 (Post 30) min after the intervention. In Experiment 2, after practicing ankle dorsiflexion movement, the maximum voluntary contraction (MVC) of each muscle was measured to evaluate the joint movement function before performing the ankle movement task. The ankle joint movement task was performed three times before and after the intervention under the same attention conditions as Experiment 1 with a 10 s rest interval. Each session was rested for at least 5 min

The experimental protocol is illustrated in Fig. 1. In Experiment 1, the stimulus intensities of the conditioned stimulus and the test stimulus were set before the RI measurement. RI was measured before (Pre), immediately after (Post), 5 min after (Post 5), 10 min after (Post 10), 20 min after (Post 20), and 30 min after (Post 30) the RPM intervention. In both experiments, we applied four attention conditions: control, forward attention, monitor attention, and ankle joint attention. In Experiment 2, the joint movement function was performed before and after the RPM intervention to examine the intervention effect on RI. Joint movement function was measured 5 min after the end of the intervention when RI enhancement is most apparent [12, 13]. A 5 min break was given between each session.

### Electromyography (EMG)

The distance between the Ag/AgCl electrodes (Blue Sensor, METS, Tokyo, Japan) for the surface electromyogram was 20 mm. The electrodes were placed on the tibialis anterior (TA) and soleus (Sol) muscles according to the surface EMG for non-invasive assessment of muscles protocols [33]. A ground electrode was placed between the electrical stimulation electrode and the TA surface electromyogram electrode [34, 35]. Electromyographic activity was filtered at a bandpass filter of 10–1000 Hz and amplified 100 × (FA-DL-720–140; 4Assist, Tokyo, Japan) before being digitally stored (10 kHz sampling rate) on a personal computer for offline analysis. Data analysis was performed using PowerLab 8/30 and LabChart 7 (both AD Instruments, Colorado Springs, CO, USA).

### RPM intervention

We adopted the RPM parameters that most effectively enhanced RI in our previous studies [12, 13]; specifically, an intervention time of 10 min, a movement speed of 160°/s, and a movement range of 30° ankle plantar flexion to 10° dorsiflexion. The participant’s thighs and feet were fixed to the seat surface and the foot plate, respectively, to maintain the lower limb position throughout the experiment (Takei Scientific Instruments, Niigata, Japan).

### Attention conditions

**Fig. 2:**
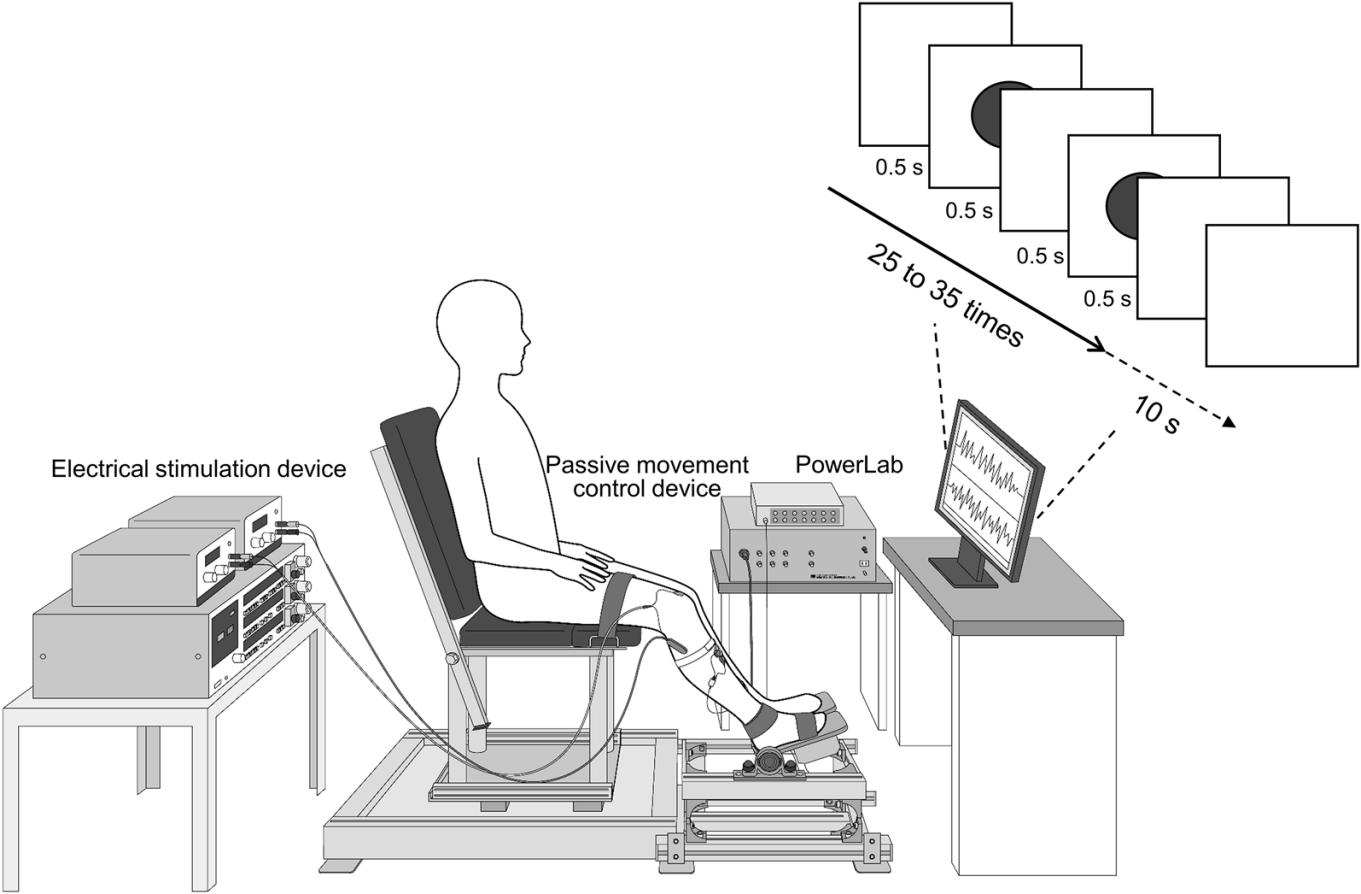
Attention condition. The diagram illustrates the monitor attention condition. Participants were asked to count the circles randomly presented on the monitor 25–35 times at 1 s intervals

Four attention conditions (control, forward attention, monitor attention, and ankle joint attention) were randomly applied during a 10 min RPM intervention, with an interval of at least 3 days between each condition. For the control condition, the participant was sat in a chair for 10 min with attention to the front wall (without RPM intervention). For the forward attention condition, the participant’s attention was directed to the anterior wall during the 10-min RPM intervention. For the monitor attention condition, the participant’s attention was directed to the monitor in front of them during the 10-min RPM intervention, and they were asked to count the circles displayed on the monitor at 1 s intervals. The circle was randomly displayed 25–35 times, hidden for 10 s, and then displayed again (Fig. 2). During the period when the circle was not displayed, the participant was asked to report to the examiner the number of times the circle was displayed while also maintaining attention to the monitor. For the ankle joint attention condition, the participant was asked to count the number of ankle joint movements during the 10-min RPM intervention. The count was taken at the time of maximum ankle joint dorsiflexion. The examiner instructed the participant when to start and pause the count. The counting time was a cycle in which the range of 25–35 s was randomly counted. The counting was then paused for 10 s and resumed at the signal of the examiner. The participant reported the number of counts to the examiner during the count pause, and the participant was instructed to maintain attention to the ankle joint during the 10-s count pause. The participants were asked to perform the count to ensure that the ankle joint was being observed [32]. The effect of the counting task was examined by comparing the monitor counting attention condition with the forward attention condition.

### RI measurement

The RI measurement method is described in detail in our previous publications [12–14]. Briefly, an electrical stimulation device (SEN-8203, Nihon Kohden, Tokyo, Japan) was used to apply electrical stimulation (1 ms duration, square wave) via an isolator (SS-104J, Nihon Kohden, Tokyo, Japan). We measured the RI of the pathway that inhibits Sol (test stimulus) from TA (conditional stimulus). The conditioned stimulus stimulated the common fibular nerve, which is the dominant nerve of TA, and the stimulus intensity was set to the M wave threshold (≤ 100 µV). The test stimulus stimulated the tibial nerve, which is the dominant nerve of Sol, and the stimulus intensity was set to achieve a Sol H-reflex amplitude value of 15–25% of the maximum M wave amplitude value (Mmax). The RI stimulation conditions were a 2 ms conditioning stimulation-test stimulation interval (CT-interval 2 ms), a 20 ms conditioning stimulation-test stimulation interval (CT-interval 20 ms), and a test stimulus without a conditioned stimulus (single). The stimulation frequency was 0.3 Hz. The CT-interval 2 ms generates the largest amount of reciprocal Ia inhibition [36, 37], and CT-interval 20 ms generates the largest amount of D1 inhibition [36].

### Joint movement function

The task movement of the joint movement function was the ankle dorsiflexion movement. After 5 min of pre-attention, the ankle dorsiflexion task was performed three times before and after each attention condition with a rest interval of 10 s between tasks. Like Experiment 1, the task movement range was from 30° ankle plantar flexion to 10° dorsiflexion. The participant was seated with their arms folded in front of their chest and instructed to quickly start the dorsiflexion with maximum effort in their own timing after the examiner’s signal.

### Data analysis

#### Experiment 1

For the RI data analysis, the peak-to-peak values of the waveform amplitudes (12 waveforms) under each stimulus condition were averaged, and the Sol H reflection amplitude value and the M wave amplitude value were calculated. The RI was calculated by dividing the Sol H reflection amplitude value by the maximum M wave amplitude value in percent notation (Sol H-reflex amplitude as the percentage of Mmax). When comparing the changes over time in each attention condition, the H-reflex amplitude value of the test stimulus with the conditioned stimulus was divided by the H-reflex amplitude value of the test stimulus and presented as a percentage ([Amplitude of conditioned H-reflex amplitude/test H-reflex amplitude] × 100).

#### Experiment 2

The analysis items for the ankle dorsiflexion task were TA and Sol EMG, co-contraction index (CI), ankle dorsiflexion peak torque (PT), and rate of joint movement development (RJD). The analysis sections are (1), from the start of TA EMG to the start of the joint movement; (2), from the start of the joint movement to the end of the joint movement; and (3), from the start of TA EMG to the end of the joint movement. The starting point of TA EMG was defined as the time point when the resting mean EMG ± 3 standard deviations (SD) was exceeded. The EMG of TA and Sol was calculated as the average EMG of each analysis range divided by the maximum voluntary contraction (MVC). The ankle dorsiflexion PT analyzed the maximum value of the ankle dorsiflexion torque of the task movement. The RJD was calculated by dividing the joint angle (40°) from the start to the end of the movement task by the time (s). The CI calculation method is as follows [38]:

$$CI=\frac{2{I}_{ant}}{{I}_{total}}\times 100\%$$

where *I*_*ant*_ is the area of the waveform on which the antagonist muscles acted, calculated using the following equation:

$${I}_{ant}={\int }_{t1}^{t2}{EMG}_{TA}\left(t\right)dt+{\int }_{t2}^{t3}{EMG}_{Sol}(t)$$

where *t1* to *t2* indicate the period in which Sol EMG is less active than TA EMG, and *t2* to *t3* indicate the period in which TA EMG is less active than Sol EMG. *I*_*total*_ is the total integral value of Sol and TA EMG calculated using the following formula:

$${I}_{total}={\int }_{t1}^{t3}[{EMG}_{TA}+{EMG}_{Sol}](t)$$

#### Statistical processing

For Experiment 1, repeated measures three-way ANOVA was performed to compare the attention condition (control, forward attention, monitor attention, ankle joint attention) × the stimulus condition (single, CT-interval 2 ms, CT-interval 20 ms) × the measurement time (Pre, Post, Post 5, Post 10, Post 20, Post 30). As a post-test, the comparison of the stimulus conditions of each attention condition was performed by applying the Bonferroni correction to the paired *t* test. To compare the measurement time of each attention condition, the Bonferroni correction was applied to the paired *t* test. For Experiment 2, paired *t* tests were used to compare the pre and post results in each analysis interval. The significance level was set to 5% for all analyses.

## Results

### Experiment 1

**Fig. 3:**
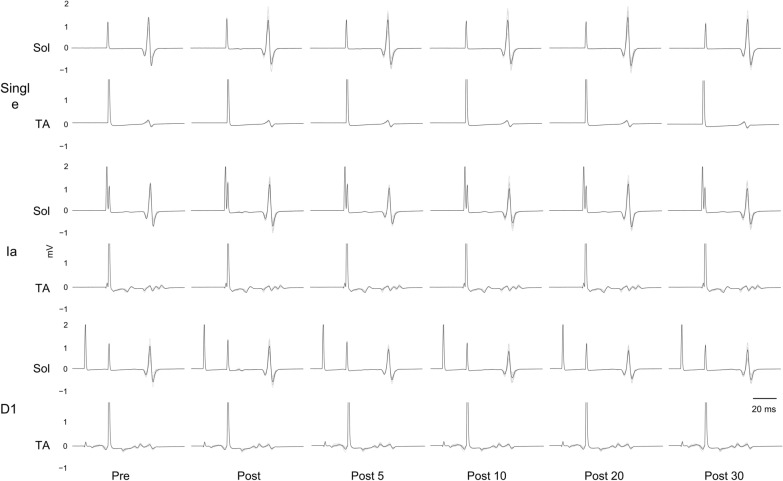
Soleus (Sol) and tibialis anterior (TA) raw data tracing. Representative raw data tracing of one participant for the ankle joint attention condition. From top to bottom, the stimulation conditions were single, conditioning stimulation-test stimulation interval (CT-interval) 2 ms (reciprocal Ia inhibition), and CT-interval 20 ms (D1 inhibition). The 12 waveforms of the Sol H-reflex are shown, and the bold black lines are the summed averages of the 12 waveforms. The horizontal data show the change over time before (Pre), immediately after (Post), and 5 (Post 5), 10 (Post 10), 20 (Post 20), and 30 (Post 30) min after the intervention

Sol background EMG, Sol Mmax amplitude values, and TA M wave amplitude values are shown in Tables 1, 2, and 3. Representative Sol and TA waveforms are shown in Fig. 3. The results of repeated measures three-way ANOVA did not detect a significant effect of the attention condition [*F*(3, 57) = 2.575, *p* = 0.063, partial *η*^2^ = 0.119], but detected significant effects of the stimulus condition [*F*(2, 38) = 176.473, *p* < 0.001, partial *η*^2^ = 0.903], the measurement time [*F*(5, 95) = 8.822, *p* < 0.001, partial *η*^2^ = 0.317], and the interaction of three factors [*F*(30, 570) = *4.426*, *p* < 0.001, partial *η*^2^ = 0.189].

**Table 1:** Soleus background electromyography (μV) for each condition before (Pre), immediately after (Post), and 5 (Post 5), 10 (Post 10), 20 (Post 20), and 30 (Post 30) min after the intervention

	Pre	Post	Post 5	Post 10	Post 20	Post 30
Control	2.8 ± 0.1	2.8 ± 0.1	2.7 ± 0.1	2.7 ± 0.1	2.7 ± 0.1	2.6 ± 0.1
Forward attention	2.7 ± 0.1	2.7 ± 0.1	2.7 ± 0.1	2.7 ± 0.1	2.7 ± 0.1	2.7 ± 0.1
Monitor attention	2.8 ± 0.1	2.7 ± 0.1	2.7 ± 0.1	2.7 ± 0.1	2.7 ± 0.1	2.7 ± 0.1
Ankle joint attention	2.8 ± 0.1	2.8 ± 0.1	2.8 ± 0.1	2.6 ± 0.1	2.7 ± 0.1	2.7 ± 0.1

Data are presented as the mean ± standard error. Soleus background EMG is the EMG 30–50 ms before the test stimulus

**Table 2:** Soleus maximum M wave amplitude values (mV) for each condition

Control	Forward attention	Monitor attention	Ankle joint attention
9.31 ± 0.77	9.40 ± 0.60	9.53 ± 0.47	9.69 ± 0.57

Data are presented as the mean ± standard error

**Table 3:** Tibialis anterior M wave amplitude values (μV) for each condition before (Pre), immediately after (Post), and 5 (Post 5), 10 (Post 10), 20 (Post 20), and 30 (Post 30) min after the intervention

	Pre	Post	Post 5	Post 10	Post 20	Post 30
Control	83.1 ± 1.5	82.7 ± 1.5	82.5 ± 1.5	83.1 ± 1.3	83.3 ± 1.4	83.1 ± 1.3
Forward attention	83.1 ± 1.4	82.9 ± 1.4	82.9 ± 1.3	83.2 ± 1.5	82.2 ± 1.8	83.5 ± 2.7
Monitor attention	82.7 ± 1.4	82.8 ± 1.4	82.2 ± 1.5	82.7 ± 1.4	82.6 ± 1.5	82.7 ± 1.4
Ankle joint attention	82.3 ± 1.5	82.0 ± 1.5	82.1 ± 1.4	82.4 ± 1.5	82.4 ± 1.5	83.2 ± 1.3

Data are presented as the mean ± standard error

There was no significant difference in the H-reflex amplitude value under the single condition at each measurement time under each attention condition (Table 4). Therefore, the change in the Sol H-reflex amplitude value with respect to the conditioned stimuli was not dependent on the test stimulus intensity.

**Table 4:** Soleus H-reflex amplitude (% of Mmax) for each condition before (Pre), immediately after (Post), and 5 (Post 5), 10 (Post 10), 20 (Post 20), and 30 (Post 30) min after the intervention

		Pre	Post	Post 5	Post 10	Post 20	Post 30
Control	single	20.4 ± 0.3	20.4 ± 0.4	20.6 ± 0.4	19.9 ± 0.4	20.5 ± 0.5	20.4 ± 0.4
	CT-interval 2 ms	17.6 ± 0.5‡	17.8 ± 0.6‡	17.8 ± 0.5‡	17.3 ± 0.5‡	17.5 ± 0.5‡	17.6 ± 0.5‡
	CT-interval 20 ms	15.2 ± 0.4‡	15.3 ± 0.4‡	15.3 ± 0.4‡	15.0 ± 0.4‡	15.3 ± 0.5‡	15.5 ± 0.4‡
Forward attention	single	20.0 ± 0.2	21.2 ± 0.4	21.2 ± 0.4	21.0 ± 0.3	20.4 ± 0.5	20.1 ± 0.4
	CT-interval 2 ms	17.3 ± 0.3‡	18.4 ± 0.5‡	17.2 ± 0.5‡	17.2 ± 0.5‡	17.6 ± 0.6‡	17.4 ± 0.5‡
	CT-interval 20 ms	15.4 ± 0.3‡	16.4 ± 0.5‡	14.9 ± 0.4‡	15.1 ± 0.4‡	14.8 ± 0.5‡	15.6 ± 0.4‡
Monitor attention	single	19.7 ± 0.3	21.5 ± 0.4	21.0 ± 0.4	20.9 ± 0.3	20.6 ± 0.3	20.0 ± 0.3
	CT-interval 2 ms	17.1 ± 0.3‡	18.9 ± 0.3‡	17.3 ± 0.4‡	17.2 ± 0.4‡	17.9 ± 0.4‡	17.6 ± 0.3‡
	CT-interval 20 ms	15.4 ± 0.3‡	17.0 ± 0.5‡	15.1 ± 0.4‡	15.1 ± 0.4‡	15.3 ± 0.4‡	15.7 ± 0.3‡
Ankle joint attention	single	21.5 ± 0.3	22.5 ± 0.2	21.5 ± 0.3	21.6 ± 0.3	20.9 ± 0.4	20.7 ± 0.3
	CT-interval 2 ms	19.2 ± 0.4‡	19.8 ± 0.4‡	17.6 ± 0.5‡	18.0 ± 0.5‡	18.3 ± 0.6‡	18.4 ± 0.4‡
	CT-interval 20 ms	16.7 ± 0.4‡	17.3 ± 0.5‡	15.1 ± 0.5‡	15.2 ± 0.5‡	15.1 ± 0.4‡	15.1 ± 0.4‡

Data are presented as the mean ± standard error. Sol H-reflex and M wave amplitude values were calculated as the mean ± standard error of the peak-to-peak values of the amplitude of each waveform. This value represents H-reflex/Mmax × 100. Data were analyzed by comparing the H-reflex amplitude value of the single condition (divided by Mmax) vs. the H-reflex amplitude value (divided by Mmax) for each of the two CT-interval conditions (2 and 20 ms). CT-interval, conditioning stimulation-test stimulation interval; Mmax, maximum M wave amplitude

^‡^*p* < 0.001 (paired *t* test with Bonferroni correction)

The single condition obtained at each measurement time of each attention condition was compared with the CT-interval conditions. The H-reflex amplitude value was significantly lower under the CT-interval 2 ms and CT-interval 20 ms conditions than under the single condition at all measurement times under all attention conditions (*p* < 0.001, Table 4). The results demonstrated that reciprocal Ia inhibition (CT-interval 2 ms) and D1 inhibition (CT-interval 20 ms) occurred under all conditions.

**Fig. 4:**
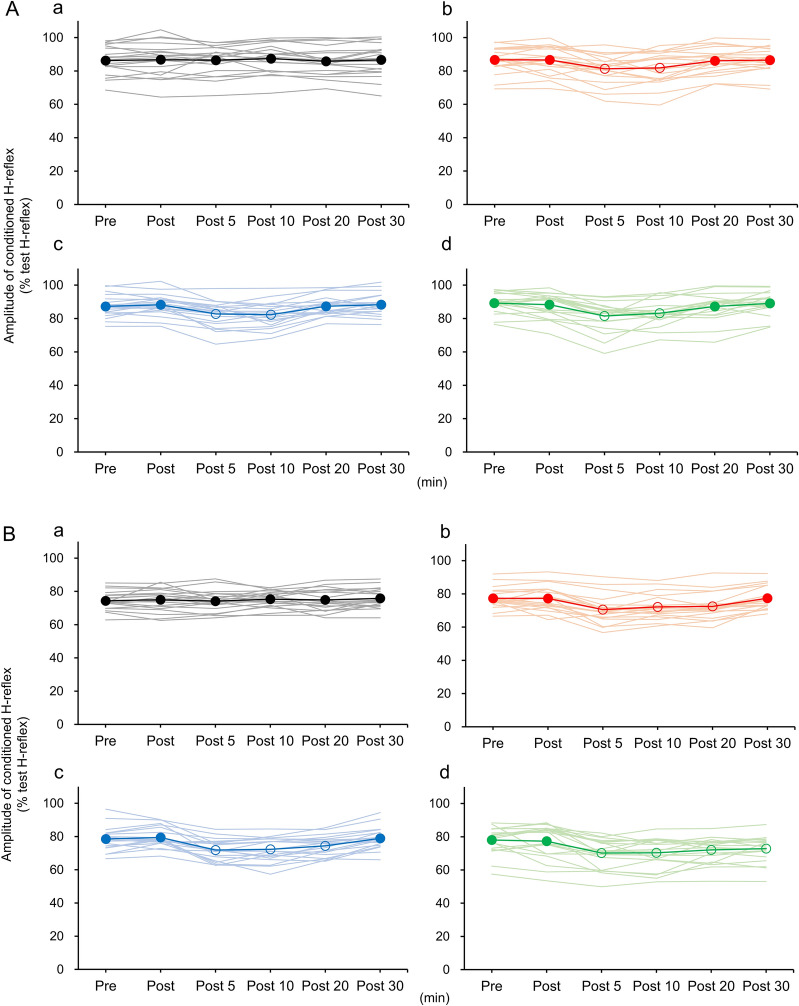
Changes in reciprocal inhibition (RI) over time. **A** shows conditioning stimulation-test stimulation interval (CT-interval) 2 ms, and **B** shows CT-interval 20 ms. The following four conditions are shown: a, control; b, forward attention; c, monitor attention; and d, ankle joint attention. The thin solid line shows the change over time in 20 participants, and the thick solid line shows the average value. The vertical axis shows the amplitude of the conditioning H-reflex/amplitude of the test H-reflex × 100. The horizontal axis shows the time points before the attention condition intervention (Pre), immediately after the intervention (Post), and 5 (Post 5), 10 (Post 10), 20 (Post 20), and 30 (Post 30) mins after the intervention. Bonferroni correction was applied to the paired *t* test for the comparison of Pre with the other measurement times. Filled circles are values that were not significantly different from Pre. Open circles are values that were significantly different from Pre (*p* < 0.05)

The H-reflex amplitude value at the measurement times under CT-interval 2 ms and CT-interval 20 ms were compared with the Pre measurement (Fig. 4A, B). Under CT-interval 2 ms, the control condition did not show a significant difference in the H-reflex amplitude value compared with Pre. In the forward attention condition, monitor attention condition, and ankle joint attention condition, the H-reflex amplitude value was significantly reduced at Post 5 and Post 10 compared with Pre (*p* < 0.001). Similarly, the H-reflex amplitude values under the control condition did not significantly differ from Pre under CT-interval 20 ms. In the forward attention condition and the monitor attention condition, the H-reflex amplitude value was significantly lower at Post 5, Post 10, and Post 20 compared with Pre (*p* < 0.001). As for the ankle joint attention condition, the H-reflex amplitude value was significantly reduced at Post 5, Post 10, Post 20, and Post 30 compared with Pre (*p* < 0.001).

### Experiment 2

**Fig. 5:**
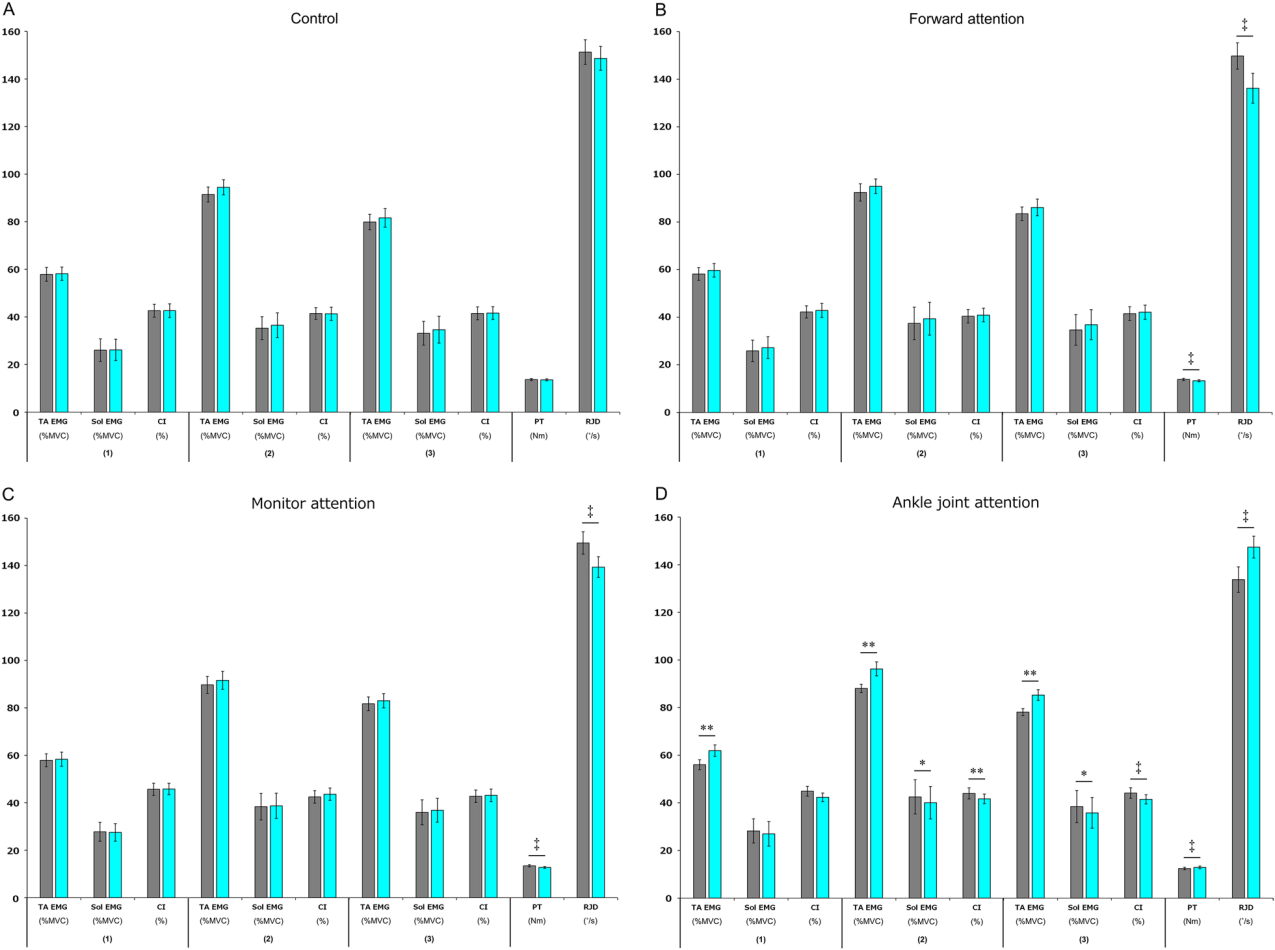
Joint movement function. **A** is the control condition, **B** is the forward attention condition, **C** is the monitor attention condition, and **D** is the ankle joint attention condition. The bar graph shows the mean ± standard error of electromyography (EMG), contraction index (CI), ankle dorsiflexion peak torque (PT), and rate of joint movement development (RJD) of the Soleus (Sol) and tibialis anterior (TA) muscles. Analysis sessions are: (1), start of TA EMG to start of joint movement; (2), start of joint movement to end of joint movement; and (3), start of TA EMG to end of joint movement. The numerical units on each vertical axis are % maximum voluntary contraction (MVC) for EMG, % for CI, Nm for ankle dorsiflexion PT, and °/s for RJD. The gray bars show the pre-intervention values (pre), and the blue bars show the post-intervention values (post). A paired *t* test was used to compare pre and post. *p < 0.05, **p < 0.01, ‡*p* < 0.001

Comparing the EMG Pre and Post results within each attention condition revealed a significant increase in EMG for TA (*p* < 0.01) and a significant decrease in EMG for Sol (*p* < 0.05) for all analysis sessions in the ankle joint attention condition (Fig. 5A–D).

Comparing the Pre and Post ankle dorsiflexion PT within each attention condition, the ankle dorsiflexion PT was significantly reduced under the forward attention condition and the monitor attention condition (*p* < 0.001). Under the ankle joint attention condition, the ankle dorsiflexion PT was significantly increased (*p* < 0.001).

Comparing the Pre and Post RJD within each attention condition, the RJD significantly decreased under the forward attention condition and the monitor attention condition (*p* < 0.001). The RJD significantly increased under the ankle joint attention condition (*p* < 0.001).

Comparing the pre- and post-CI within each attention condition, the CI decreased significantly in the analysis sessions of ankle joint attention condition (2) (*p* < 0.01) and (3) (*p* < 0.001).

## Discussion

The results of this study demonstrated that the duration of RI enhancement could be extended by focusing the participant’s attention on the ankle joint targeted during the RPM RI enhancement intervention. The joint movement function evaluation revealed that the joint movement function decreased with RPM intervention alone and improved when the participant focused their attention on the ankle joint during the RPM intervention.

In this study, we instructed the participant to perform a counting task to confirm that the participant’s attention was focused on the target. The results of Experiment 1 and Experiment 2 showed that the effects during the counting of ankle joint movements (ankle joint attention condition) were similar to those of the forward attention condition and the monitor attention condition, which involved counting the appearances of a circle on the monitor, indicating the effect of counting was negligible. This finding is consistent with the results of a previous report [32], who showed that the effect of counting on an attention task during index finger RPM was extremely small.

In this study, the RI enhancement effect of RPM intervention supported the results of our previous study [12, 13], i.e., reciprocal Ia inhibition is enhanced for 10 min after the intervention, and D1 inhibition is enhanced for 20 min after the intervention, demonstrating that RPM is a useful intervention method for RI enhancement. The present study revealed that the participant’s attention to the RPM intervention target (the ankle joint) extended the RI enhancement for up to 30 min after the RPM intervention, supporting our hypothesis. The effect of RPM alone on RI is attributed to the repeated muscle lengthening and shortening, which increase the firing frequency of afferent Ia fibers from muscle spindles in the TA and activate RI-inhibitory interneurons, contributing to the enhancement of RI. Similar results have been obtained using PES [15, 17, 21, 23, 24], a technique that specifically stimulates Ia fibers to enhance RI.

In addition, the activity of motor-associated cortical areas activates RI-inhibitory interneurons and contributes to RI enhancement, and the intervention effect of peripheral stimulation is enhanced by brain stimulation [14, 16–18, 21, 23]. The combination of brain stimulation and peripheral stimulation is effective for RI enhancement in RI-inhibitory interneurons via the convergent input from the motor cortex and the afferent fibers of TA [39–42]. In this study, as an alternative to brain stimulation, we focused the participant’s attention on the intervention target during RPM to easily increase motor cortex excitability. A previous study by our research group [32] showed that attention to the intervention target during RPM increased the corticospinal tract excitability of the target muscle. The increase in corticospinal tract excitability caused by attention to the target has been attributed to the inhibition of SICI and short-latency afferent inhibition (SAI) [43, 44]. The results of the present study suggest that the factors contributing to RI enhancement when attention was focused on the RPM intervention target may include motor cortex activation, an increase in the descending input, and RI-inhibitory interneuron activation, which prolonged the post-intervention RI enhancement effect. Previous studies have shown that motor imagery is an effective intervention method for activating the motor cortex. The RI enhancement effect achieved using motor imagery during PES intervention was comparable to that achieved using peripheral stimuli with attention directed to the target [25]. Based on the findings of these previous studies, attention to the intervention target during RPM enhanced RI and prolonged the post-intervention effect to 30 min, which is the longest post-intervention effect achieved with the shortest intervention time (10 min) among the RI-enhancing methods that have been investigated. These findings will facilitate the development of an effective and efficient intervention method for clinical application.

The joint movement function evaluation in Experiment 2 revealed that the joint movement function decreased under the forward attention condition and the monitor attention condition and was improved under the ankle joint attention condition. In addition, this is the first study to evaluate joint movement function as a post-intervention effect of the RPM RI enhancement method. In the forward attention and monitor attention conditions, the RI was increased by the RPM intervention, but the joint movement function decreased, contrary to our hypothesis. However, under the ankle joint attention condition, the RI was increased by the RPM intervention, and the joint movement function was improved, supporting our hypothesis. The reason for the decrease in joint movement function under the forward attention and monitor attention conditions could be the decrease in the corticospinal tract excitability of the agonist muscle, which may be caused by post-exercise depression (PED). It has been reported that PED is induced by non-fatigue active movement and passive movement [45–51]. PED is caused by the frequent repetition of activity in the primary motor area (M1) and induced by increased SICI, a measure of GABAergic intracortical inhibitory circuit excitability [50–52]. It has been reported that SICI is modulated by input from the proprioceptors [53]. The results of this study suggest that RPM-induced proprioceptor activity increased SICI, inhibited M1 excitability, and decreased the joint movement function.

Under the ankle joint attention condition, TA EMG increased during dorsiflexion after RPM intervention, the CI decreased due to the decrease in Sol EMG, and the joint movement function improved due to the increase in ankle dorsiflexion PT and RJD. The results of this study suggest that in addition to the RI enhancement (antagonist muscle EMG decrease during joint movement) by RPM, attention to the RPM intervention target may have increased M1 activity and thus the muscle output of the main movement muscle, improving the joint movement function. In a previous study [32], focusing attention on the index finger during RPM of the index finger increased the MEP and the excitability of the corticospinal tract. Corticospinal tract excitability is caused by a decrease in SAI due to attention [44] because SAI modulates the effect of somatosensory input on motor cortex excitability [54]. Studies have shown that SICI decreases with attention [43], and SICI is involved in the GABAergic inhibition mechanism [55, 56]. Therefore, attention to the ankle joint during RPM may have increased the excitability of M1 by decreasing the excitability of intracortical inhibitory circuits, which activated the main action muscle (TA) during the ankle dorsiflexion task and inhibited the antagonist muscle (Sol) by enhancing RI. Thus, the joint movement function was improved by decreasing CI and increasing ankle dorsiflexion PT.

One limitation of this study was that the excitability of the corticospinal tract corresponding to the muscle targeted for RPM intervention could not be evaluated. However, in previous studies [32, 52, 57], the excitability of corticospinal tracts and spinal anterior horn cells after RPM was evaluated and examined under many parameters, including different speeds and numbers of RPM movements and attention, and we believe that the findings support the results of this study. The results of the joint movement function evaluation in this study suggest that similar corticospinal tract excitability results were obtained during ankle joint RPM as in the previous study.

### Clinical application

RPM is an effective and efficient RI enhancement intervention method. In addition, the findings of this study suggest that simply focusing attention on the RPM intervention target can enhance RI potentiation and improve joint movement function, which may be an effective adjunct therapy for functional recovery after central nervous system injury. RI-enhancing methods other than RPM have only shown effects for 10 min after interventions of 15 min or longer [14–23]. In this study, the participant was asked to focus their attention on the intervention target during RPM, and a 10 min intervention resulted in a 30-min RI enhancement. Prolonging the duration of the intervention effect using adjunct therapy may improve rehabilitation. Future studies should investigate the post-intervention effect and joint movement function, gait, and balance function in patients.

## Conclusions

In this study, we examined RI and joint movement function after RPM intervention, during which the participant’s attention was focused on different targets. As a result, it was clarified that the RPM intervention effect could be improved by focusing the participant’s attention on the intervention target (in this case, the ankle joint) during the 10-min RPM. Furthermore, the RI enhancement lasted for 30 min after the intervention, and the joint movement function improved.

## Data Availability

The data that support the findings of this study are available from the corresponding author, [R.H.], upon reasonable request.

## Abbreviations

CI: Contraction index
MVC: Maximum voluntary contraction
PED: Post-exercise depression
PES: Patterned electrical stimulation
PT: Peak torque
RI: Reciprocal inhibition
RJD: Rate of joint movement development
RPM: Repetitive passive movement
SAI: Short-latency afferent inhibition
SD: Standard deviations
SICI: Short-interval intracortical inhibition
TA: Tibialis anterior
